# Low-Friction and Corrosion-Resistant Orthodontic Stainless Steel Archwires with Functional Carbon Films

**DOI:** 10.3390/nano15211615

**Published:** 2025-10-23

**Authors:** Pengfei Wang, Minghui Hao, Shiqi Cheng

**Affiliations:** Institute of Nanosurface Science and Engineering (INSE), State Key Laboratory of Radio Frequency Heterogeneous Integration, College of Mechatronics and Control Engineering, Shenzhen University, Shenzhen 518060, China; 17625797242@163.com (M.H.); chengshiqi0212@163.com (S.C.)

**Keywords:** orthodontic archwire and bracket, carbon film, graphene sheets, fluoride toothpaste, corrosion, peeling, friction and wear

## Abstract

To mitigate the adverse effects of immersion in fluoride-containing solutions on the surface corrosion of orthodontic stainless steel archwires, carbon films were fabricated on these archwires under various deposition times and substrate bias voltages using a self-designed plasma sputtering system. Structural analysis revealed that the carbon films deposited at lower substrate bias voltages were classified as amorphous carbon films, whereas those fabricated at higher substrate bias voltages were identified as graphene nanocrystalline carbon films. Particularly, immersion tests and electrochemical experiments demonstrated that carbon film prepared at a substrate bias voltage of +50 V for 80 min exhibited exceptional corrosion resistance. Furthermore, a low friction coefficient and low wear rate were obtained even after soaking in a fluoride toothpaste mixed solution. The mechanisms underlying the corrosion resistance and friction properties of these superior carbon films were thoroughly investigated. This study provides valuable insights into the application of carbon film for reducing friction and wear while enhancing corrosion resistance, thus promoting their practical clinical applications in coated orthodontic stainless steel archwires.

## 1. Introduction

Malocclusion is one the three most common oral diseases in clinical application (global prevalence of 56% [1,2,3,4,5]), which not only significantly affects the facial aesthetics of patients, but also affects their mental health and functionality of the oral and maxillofacial systems. Nowadays, clinical orthodontic treatment serves as a prevalent method for correcting malocclusion. A metallic fixed appliance, which consists of brackets bonded to the teeth and archwires that connect the brackets, is designed to align teeth and restore the occlusal relationship by precisely controlling the three-dimensional tooth movement [6,7,8,9,10,11,12].

The oral cavity is a highly complex biological environment. Temperature fluctuations, pH changes, high humidity, mechanical stress, and the presence of microorganisms in the oral environment all make it highly conducive to the promotion of the corrosion of metallic orthodontic appliances, with electrochemical corrosion being the main type [13,14,15,16]. Fluorine, as an active chemical element, demonstrates a pronounced anti-caries effect, on the one hand, while on the other hand, it could induce severe corrosion damage in metallic materials, thus accelerating material degradation and metal ion release [17,18,19,20,21].

The corrosion resistance of the archwires and brackets has been significantly enhanced through the application of surface coatings, such as metallic layers, metal oxides, polymers, and diamond-like carbon (DLC) [22,23,24]. Specifically, Ryu et al. [25] incorporated platinum (Pt) into silver (Ag) films by using the physical vapor deposition technique to enhance the chemical stability. Potentiodynamic polarization tests revealed that the corrosion resistance of the AgPt-film-coated stainless steel archwires was significantly improved with the Pt content reaching up to 7%. Wu et al. [26] deposited Al–SiO_2_ composite coatings onto the surfaces of the NiTi and stainless steel archwires using a magnetron sputtering technique. The surface smoothness and overall corrosion resistance of the materials were strongly enhanced, while exhibiting no significant cytotoxicity. Espinar et al. [27] achieved a nickel-free surface through a novel oxidation treatment method. The generated titanium oxide layer markedly improved the corrosion resistance of the NiTi archwires and suppressed the release of nickel ions. Zhang et al. [28] deposited DLC coatings onto the surface of stainless steel archwires using plasma-enhanced chemical vapor deposition (PECVD), and applied salt bath nitrocarburizing to achieve surface hardening of the archwires. It was found that both DLC coating and nitrocarburizing treatment significantly enhanced surface hardness, reduced the friction coefficient, and exhibited favorable biocompatibility. A reduction in corrosion current density by two orders of magnitude was also observed for the DLC-coated archwire. Sui et al. [29] fabricated DLC coatings onto the surface of the NiTi archwires using an ion implantation technique and clarified that the DLC coating not only significantly improved the corrosion resistance of the NiTi archwires but also effectively inhibited the release of nickel ions. Kobayashi et al. [30] immersed both DLC-coated and uncoated NiTi archwires in physiological saline at 37 °C for a duration of up to six months and assessed the cytotoxic effects of nickel ion release using squamous carcinoma cells. It was revealed that the DLC coating reduced the nickel ion concentration in the solution by one-sixth, indicating its role as a diffusion barrier in corrosive environments and confirming its favorable biocompatibility. Fróis et al. [31] deposited the a-C:H coatings on the SS316L substrate with a Cr-based interlayer. The common corrosion, delamination, and coating detachment from the substrates could not be observed after a 30-day immersion test in artificial saliva solution. However, it is still a great challenge for fabricating an exceptional functional coating that could satisfy various functions, such as low friction, wear resistance, corrosion resistance, and even biocompatibility.

In our previous work, graphene nanocrystalline films were deposited onto stainless steel archwires to reduce the friction coefficients of the archwire–bracket sliding contacts in artificial saliva environment [32,33]. Therefore, the main objective of this work was to achieve low friction, low wear rate, and corrosion resistance of stainless steel archwires with outstanding carbon film for potential clinical applications. Versatile carbon films were fabricated on the working surfaces of the stainless steel archwires to mitigate the adverse effects of fluoride-containing solution immersion on the surface corrosion of the stainless steel archwires. Subsequently, friction and wear experiments as well as electrochemical analyses were conducted. Finally, the stainless steel archwires were characterized, and the corrosion prevention mechanism as well as low friction mechanism were systematically discussed.

## 2. Materials and Methods

A commercial AISI 304 stainless steel archwire (06Cr19Ni10) with rectangular cross-sectional dimensions of 0.017 inch × 0.025 inch (0.42 mm × 0.64 mm) (HXH-0023, Xihubiom, Hangzhou, China) was applied. The orthodontic archwire was engaged in an AISI 304 stainless steel bracket (0Cr18Ni9) with a slot size of 0.022 inches (0.56 mm) (ALS-0008, Alice Dental, Hangzhou, China). Prior to being loaded into the deposition chamber, the archwire and bracket were sequentially cleaned in an ultrasonic bath (M2800-C, BRANSON, Shanghai, China) using acetone, ethanol, and deionized water, each for a duration of 20 min.

A photograph of the customized mirror confinement electron cyclotron resonance (MCECR) plasma sputtering apparatus is shown in Figure S1. Detailed information on the ECR sputtering system has been reported in our previous studies [34,35]. The main working principle involves the formation of electron cyclotron oscillation acceleration under the control of a mirror confined magnetic field (875 G). When a positive bias voltage is applied to the substrate, the oscillating electrons are attracted to irradiate the substrate surface, thus enabling the deposition of the carbon films under the low energy electron irradiation working mode. The detailed parameters for preparing the carbon films on the surface of stainless steel archwires are presented in Table S1.

To evaluate the corrosion resistance of carbon-film-coated archwires, the specimens were soaked in a fluoride toothpaste mixed solution (i.e., fluoride toothpaste of 10 g and deionized water of 30 mL) for 16 h [36]. The working surface of the archwire was carefully observed using an optical microscope (LV-150N, NIKON, Tokyo, Japan) and a scanning electron microscope (SEM, Scios, FEI, Hillsboro, OR, USA) before and after the immersion test.

**Figure 1:**
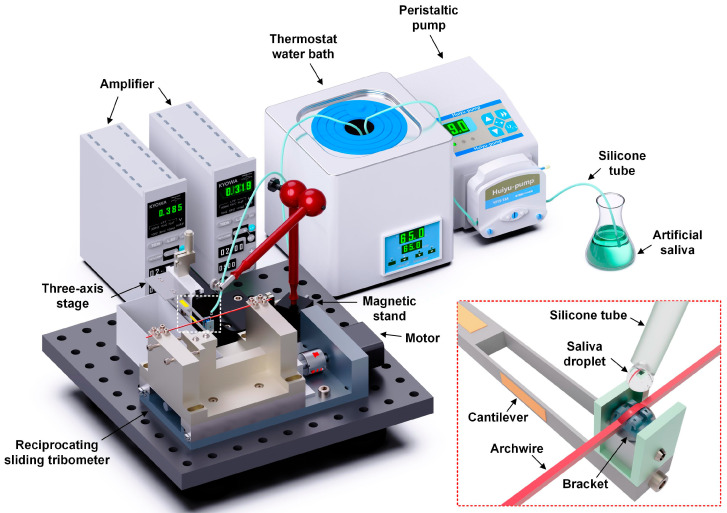
Schematic illustration of the orthodontic stainless steel archwire–bracket reciprocating sliding tribometer. Inset shows an enlarged diagram of the sliding contact of the archwire and bracket.

A custom-designed tribo-system was developed and constructed to assess the friction and wear behavior of orthodontic archwire–bracket contact combinations under an artificial saliva environment, as schematically illustrated in Figure 1. Comprehensive details regarding the structure of the tribo-system have been reported in our previous studies [32,33]. The system primarily comprised an electrically controlled displacement platform, an archwire fixation and pre-tensioning module, a bracket fixation unit, a three-axis stage, a data acquisition system, a thermostatic water bath, and a peristaltic pump. During the experimental procedure, the bracket was secured to a cantilever beam connected to the three-axis stage, while the archwire was actuated by a stepper motor-driven displacement platform, which induced reciprocating motion in the archwire fixation module. Concurrently, strain signals produced during the sliding process were captured by the strain gauge affixed to the cantilever beam and transmitted in real time via the data acquisition system to the computer for continuous recording. The peristaltic pump delivered commercial artificial saliva (ISO.10271.2011, pH = 6.9, dripping speed of 3.6 mL/min), preheated to a controlled temperature, onto the contact interface between the archwire and the bracket. The temperature of the droplet artificial saliva at the contact interface was maintained at approximately 37 °C, closely simulating the physiological temperature of the human oral cavity. The tribological experiment was conducted at a tensile force of 10 N, a normal load of 1 N, a reciprocating displacement of 0.15 mm, a sliding speed of 0.19 mm/s, and a sliding period of 5000 cycles.

The nanostructures of the deposited carbon films were examined using a double spherical aberration corrected transmission electron microscope (TEM, Titan3 Cubed Themis G2 300, THERMO FISHER, Hillsboro, OR, USA) operated at an accelerating voltage of 80 kV. The bonding structure configuration of the fabricated carbon film was obtained using a Raman spectroscope (LabRAM HR Evolution, HORIBA, Kyoto, Japan).

A 3D laser confocal microscope (VK-X250K, KEYENCE, Osaka, Japan) was applied to measure the surface roughness of the carbon-film-coated archwires (scanning area of 600 mm × 300 mm) and observe the wear scars on the coated archwires (scanning area of 200 mm × 150 mm) with a depth resolution of 0.2 mm. The observed raw data were evaluated with a standard F-filter filtering process under a cut-off value of 80 mm. The worn surfaces of the archwires were analyzed using an optical microscope and an SEM equipped with an energy dispersive X-ray spectroscope (EDS, THERMO FISHER, Hillsboro, OR, USA).

The electrochemical experiments were performed on an electrochemical workstation (Reference 600+, Gamry, PA, USA). A standard three-electrode cell configuration was established, comprising a platinum wire as the counter electrode (CE), a saturated Ag/AgCl electrode serving as the reference electrode (RE), and the surface of carbon-film-coated archwire as the working electrode (WE). The corrosion solution was a specially prepared fluoride toothpaste mixed solution (the mass ratio of fluoride toothpaste to deionized water is 1:3). In the potentiodynamic polarization test, the measured data were imported into Gamry Echem Analyst software (Version 7.05) to obtain the Tafel curve. A suitable linear region of the curve was selected for Tafel extrapolation to determine the corrosion potential and corrosion current density of the archwire.

## 3. Results

### 3.1. Characterization of the Carbon-Film-Coated Archwires

**Figure 2:**
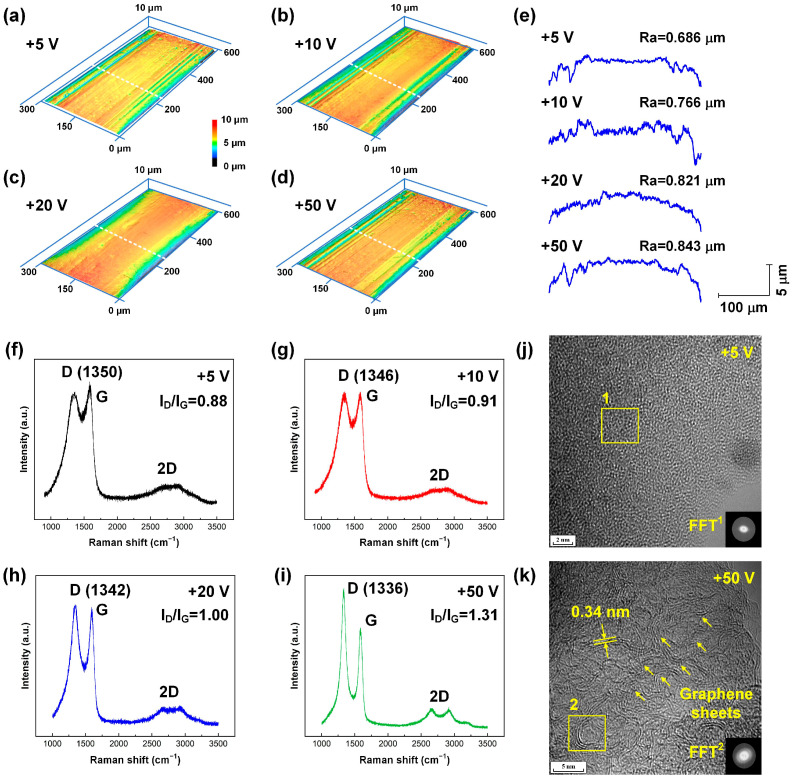
Characterization of the as-deposited carbon films prepared under various substrate bias voltages. Three-dimensional laser microscopy image of the carbon film fabricated at a substrate bias voltage of (**a**) +5 V, (**b**) +10 V, (**c**) +20 V, and (**d**) +50 V. (**e**) Two-dimensional cross-sectional profiles of the fabricated carbon films from the selected positions marked with white dotted lines in the corresponding optical images. Raman spectrum of the carbon film fabricated at a substrate bias voltage of (**f**) +5 V, (**g**) +10 V, (**h**) +20 V, and (**i**) +50 V. Plan-view HRTEM images of the carbon film fabricated at a substrate bias voltage of (**j**) +5 V and (**k**) +50 V. Insets in (**j**) and (**k**) show FFT images of the marked regions in the corresponding TEM images.

The surface characterization of the as-deposited carbon films prepared under various substrate bias voltages is summarized in Figure 2. The surface morphology and roughness profiles of the carbon-film-coated stainless steel archwires under different substrate bias voltages are shown in Figure 2a–e. Clearly, the surface roughness (Ra) of the stainless steel archwire increased gradually from 0.686 μm to 0.843 μm with an increase in substrate bias voltage from +5 V to +50 V. The increase in surface roughness could be attributed to the increased electron irradiation intensity associated with higher substrate bias voltage. Stronger electron irradiation promoted the growth of nanoscale graphene sheets, leading to a larger grain size, as described in previous studies [35].

Structural information on the carbon film was further characterized using Raman spectroscopy, as shown in Figure 2f–i. Specifically, the D peak corresponded to the degree of disorder in *sp*^2^ hybridized carbon bonding, and the G peak represented the vibrational modes associated with the *sp*^2^-bonded carbon network. The appearance of the 2D peak distinctly indicated the existence of graphitic or graphene-like structures [37]. Particularly, higher substrate bias voltage led to sharpening of both the D and G peaks, suggesting an enhancement in the crystallinity of the carbon film. Moreover, the progressive intensification of the 2D peak implies a structural evolution from a disordered to a more ordered arrangement of carbon atoms [38]. The I_D_/I_G_ ratio increased straightly from 0.88 to 1.31 with the increase in substrate bias voltage from +5 V to +50 V. Contrastingly the D peak position decreased continuously from 1350 cm^−1^ to 1336 cm^−1^ with the increase in the substrate bias voltage from +5 V to +50 V. Thus, the carbon films deposited at +5 V and +10 V were clarified as amorphous carbon films, whereas those deposited at +20 V and +50 V were identified as graphene nanocrystalline carbon films. Furthermore, high-magnification TEM imaging revealed that the carbon film prepared under a substrate bias voltage of +5 V exhibited a highly disordered state, lacking any distinct structural features, as evidenced by the dispersive ring pattern observed in the Fourier transformed filtering (FFT) image, as shown in Figure 2j. In contrast, the carbon film fabricated under a substrate bias voltage of +50 V displayed a well-defined layered crystalline structure, characterized by two distinct points in the FFT image. The interplanar spacing was measured to be approximately 0.340 nm, as illustrated in Figure 2k.

### 3.2. Immersion Test of the Carbon-Film-Coated Archwires

**Figure 3:**
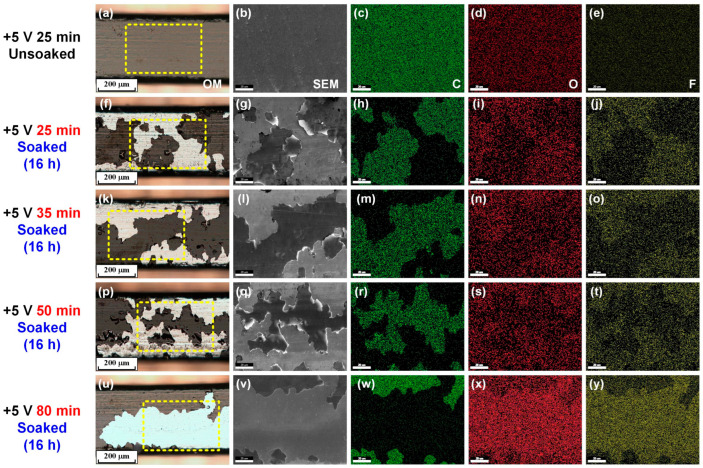
Optical images (**a**,**f**,**k**,**p**,**u**) and corresponding SEM–EDS images ((**b**–**e**,**g**–**j**,**l**–**o**,**q**–**t**,**v**–**y**), marked with yellow rectangular dotted line) of the carbon-film-coated stainless steel archwires after soaking in a fluoride toothpaste mixed solution for 16 h. (**f**–**j**) +5 V & 25 min, (**k**–**o**) +5 V & 35 min, (**p**–**t**) +5 V & 50 min, and (**u**–**y**) +5 V & 80 min. The unsoaked results of the carbon-film-coated stainless steel archwire (+5 V & 25 min) are also shown in (**a**–**e**) for reference. The scale bar in the SEM–EDS image is 20 mm.

Firstly, the stainless steel archwires were coated with carbon films fabricated under a substrate bias voltage of +5 V and deposition time varied from 25 min to 80 min (thereafter denoted as +5 V & 25 min, +5 V & 35 min, +5 V & 50 min, and +5 V & 80 min), and subsequently soaked in a fluoride toothpaste mixed solution for 16 h. After the immersion test, the surface morphology of the archwires was observed using an optical microscope and an SEM–EDS. Figure 3 presents the optical image and corresponding SEM–EDS analysis results of the coated stainless steel archwires. Severe peeling of the carbon film from the stainless stee archwire occurred after soaking in mixed solution for 16 h, and the peeling phenomenon of the carbon films mainly occurred at the edges of the stainless steel archwire, as typically shown in Figure 3f,k,p,u. The detachment of the carbon film from the archwire surface resulted in the depletion of the carbon (C) element (e.g., Figure 3h), and the enrichment of the oxygen (O, e.g., Figure 3i) and fluorine (F, e.g., Figure 3j) elements. It was also found that the extent of the peeling from the archwire surface gradually decreased with the increase in deposition time from 25 min to 80 min. The great change in element distribution was mainly attributed to the direct exposure of the stainless steel archwire to the fluoride toothpaste solution after the peeling of the carbon film, thus triggering the corrosion of fluoride ions on the stainless steel [23,39]. Therefore, more attention was paid to further optimize the deposition parameters to improve the corrosion resistance and long-term stability of the carbon film in a fluoride-containing environment.

**Figure 4:**
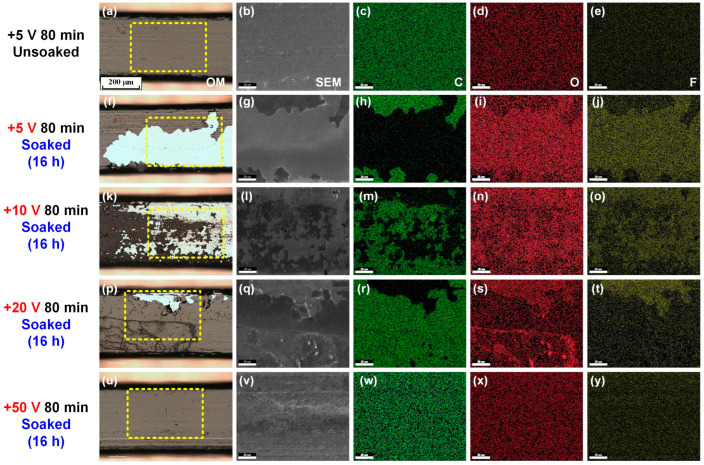
Optical images (**a**,**f**,**k**,**p**,**u**) and corresponding SEM–EDS images ((**b**–**e**,**g**–**j**,**l**–**o**,**q**–**t**,**v**–**y**), marked with yellow rectangular dotted line) of the carbon-film-coated stainless steel archwires after soaking in fluoride toothpaste mixed solution for 16 h. (**f**–**j**) +5 V & 80 min, (**k**–**o**) +10 V & 80 min, (**p**–**t**) +20 V & 80 min, and (**u**–**y**) +50 V & 80 min. The unsoaked results of the carbon-film-coated stainless steel archwire (+5 V & 80 min) are also shown in (**a**–**e**) for reference. The scale bar in SEM–EDS image is 20 mm.

Figure 4 presents the optical images and corresponding SEM–EDS analysis results of the coated stainless steel archwires with carbon film prepared under the deposition time of 80 min and various substrate bias voltages (+5 V to +50 V, and thereafter denoted as +5 V & 80 min, +10 V & 80 min, +20 V & 80 min, +50 V & 80 min) and subsequently soaked in a fluoride toothpaste mixed solution for 16 h. The degree of the carbon film peeling from the stainless steel archwire after the long-term immersion test was relatively lower than that shown in Figure 3. Moreover, the extent of carbon film peeling after soaking in the fluoride toothpaste mixed solution gradually diminished with the increase in the substrate bias voltage from +5 V to +50 V. At a substrate bias voltage of +20 V, only minor peeling occurred at the edges of the carbon film, as shown in Figure 4p, whereas at a substrate bias voltage of +50 V, the carbon film remained largely intact on the stainless steel archwire, as shown in Figure 4u, exhibiting excellent protective performance. With the mitigation of the peeling degree of the carbon film on the archwire, the distribution of carbon element greatly increased (e.g., Figure 4w) followed by a strong decrease in fluoride element (e.g., Figure 4y) on the archwire. Therefore, it could be concluded that the presence of the GSEC film significantly minimized the corrosive effects of the fluoride-containing solution on the stainless steel archwire.

### 3.3. Friction Behavior of the Carbon-Film-Coated Archwires

**Figure 5:**
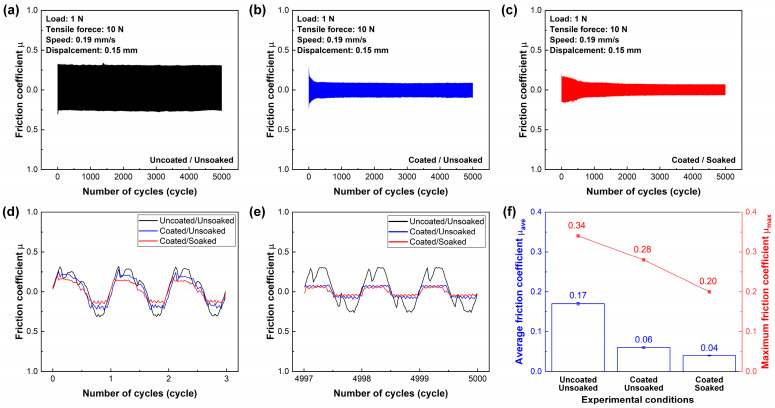
Friction behaviors of the reciprocating sliding contacts of three types of stainless steel archwires and brackets in artificial saliva environment. The carbon film was deposited onto the stainless steel archwire surface at a substrate bias voltage of +50 V and a deposition time of 80 min. The term “soaked” means the carbon-film-coated stainless steel archwire was soaked in a fluoride toothpaste mixed solution for 16 h. (**a**) Typical friction curve of the uncoated and unsoaked stainless steel archwire. (**b**) Typical friction curve of the carbon-film-coated and unsoaked stainless steel archwire. (**c**) Typical friction curve of the carbon-film-coated and soaked stainless steel archwire. (**d**) Typical friction curve of the initial three cycles (i.e., 1–3). (**e**) Typical friction curve of the final three cycles (i.e., 4997–4999). (**f**) Maximum static friction coefficients and stable average friction coefficients.

The friction curves and corresponding friction coefficients of the uncoated and unsoaked archwire (thereafter denoted as uncoated/unsoaked), coated and unsoaked archwire (thereafter denoted as uncoated/unsoaked), as well as coated and soaked archwire (thereafter denoted as uncoated/unsoaked) sliding against stainless steel bracket under artificial saliva environment are shown in Figure 5. The uncoated/unsoaked archwire exhibited a relatively high and stable friction coefficient throughout the entire sliding friction test, as shown in Figure 5a. On the contrary, the coated/unsoaked archwire exhibited a fast running-in process and then stabilized after approximately 25 cycles, as shown in Figure 5b. Similarly, the coated/soaked archwire maintained a stable stage after 75 cycles of the running-in process, as shown in Figure 5c. The above results definitely showed that lower friction coefficients were observed with the carbon-film-coated archwire, regardless of the soaking process. Specifically, at the early stage, the friction curve of the uncoated archwire remained at a relatively high level compared to the coated archwires, although the difference was minor, as shown in Figure 5d. However, at the final stage, the friction coefficient of the uncoated archwire became significantly higher than that of the coated archwire, which maintained a consistently stable friction coefficient for the whole test, as shown in Figure 5e.

The maximum static friction coefficient and average friction coefficient of three types of sliding contact combination are summarized in Figure 5f. The maximum static friction coefficient and average friction coefficient of 0.34 and 0.17 were observed with the uncoated/unsoaked archwire, respectively. Interestingly, the lowest maximum static friction coefficient and average friction coefficient of 0.20 and 0.04 were obtained with the coated/soaked archwire, respectively. There was no degradation of the frictional performance of the carbon-film-coated archwire after the soaking treatment; nevertheless, the friction coefficient was slightly decreased from 0.06 to 0.04. Hence, the carbon-film-coated archwire fabricated under a substrate bias voltage of +50 V and a deposition time of 80 min not only exhibited excellent corrosion resistance, but also retained friction-reducing property even after the long-term exposure to a fluoride-containing environment.

**Figure 6:**
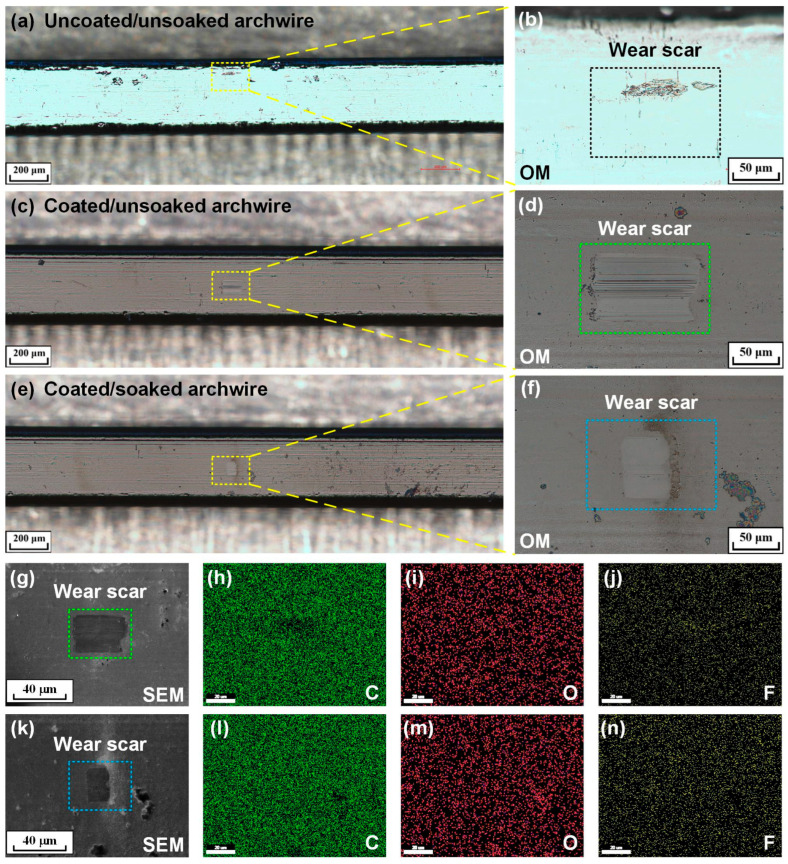
Optical images and SEM–EDS images of the worn surfaces on the stainless steel archwires after running against stainless steel brackets in an artificial saliva environment. (**a**,**b**) Wear scar on the uncoated/unsoaked archwire. (**c**,**d**,**g**–**j**) Wear scar on the coated/unsoaked archwire. (**e**,**f**,**k**–**n**) Wear scar on the coated/soaked archwire. (**a**–**f**) Optical images and (**g**,**k**) SEM images of the wear scars on the archwires. (**h**,**l**) Carbon element, (**i**,**m**) oxygen element, and (**j**,**n**) fluorine element distributions of the wear scars on the carbon-film-coated stainless steel archwires. The scale bar in EDS image is 20 mm.

Optical images of the wear scars on three types of stainless steel archwires after sliding against stainless steel brackets in an artificial saliva environment are shown in Figure 6a–f. It was clearly observed that carbon film still covered the whole wear scar on the coated stainless steel archwire, regardless of soaking or not, after running against a stainless steel bracket in an artificial saliva environment, as shown in Figure 6d,f. The peeling off or detachment of the carbon film from the archwires could not be found for the soaked carbon film. Moreover, the SEM–EDS images (i.e., Figure 6g–n) of the wear scar on the carbon-film-coated stainless steel archwires further confirmed that the carbon film did not wear out after the reciprocating sliding friction test in an artificial saliva environment. Mild wear of the carbon film was achieved in an artificial saliva environment whether soaked or not in a fluorine-containing environment. With respect to the wear scar dimensions, the width of the wear scars remained consistent for both soaked and unsoaked carbon-film-coated archwires; however, the length of the wear scar on the unsoaked archwire (i.e., Figure 6d) was approximately twice that of the soaked one (i.e., Figure 6f). Moreover, mild wear was clearly observed on the wear tracks of the brackets, as shown in Figure S2.

## 4. Discussion

### 4.1. Electrochemical Corrosion of the Carbon-Film-Coated Archwires

**Figure 7:**
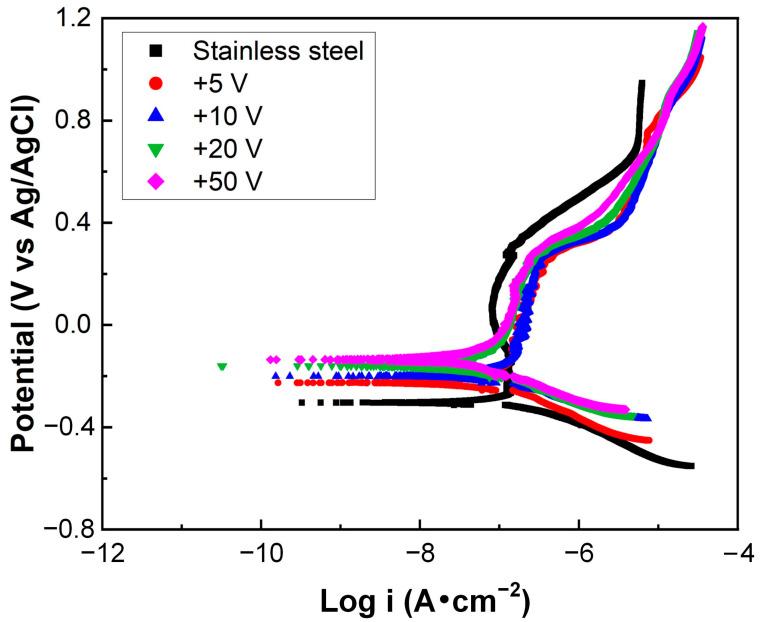
Polarization curves of the uncoated and carbon-film-coated stainless steel archwires in a fluoride toothpaste mixed solution.

Figure 7 presents the polarization curves of the carbon-film-coated stainless steel archwires prepared under different substrate bias voltages, which exhibit distinct corrosion behaviors in a fluoride toothpaste mixed solution. Detailed electrochemical corrosion parameters are summarized in Table 1. It was clarified that the carbon film deposited at the substate bias voltage of +50 V exhibited the highest corrosion potential (−135.1 mV), whereas the carbon film deposited at the substrate bias voltage of +5 V showed the lowest value (−226.3 mV), both significantly higher than that of the uncoated stainless steel archwire (−303.1 mV). Regarding corrosion current density, the highest value (0.229 μA/cm^2^) was observed for the +5 V specimen, while the lowest value (0.072 μA/cm^2^) was recorded for the +50 V specimen, all of which were lower than the uncoated stainless steel archwire (0.336 μA/cm^2^). A higher corrosion potential together with a lower corrosion current density was obtained with the increase in the substrate bias voltage from +5 V to +50 V. These findings indicated that increasing the substrate bias voltage led to a gradual increase in corrosion potential and a corresponding decrease in corrosion current density, thus demonstrating the enhanced corrosion resistance of the carbon-film-coated stainless steel archwires. Moreover, in the previous studies of corrosion resistance of stainless steel archwire, the potential and current density of AISI 304 stainless steel (18% Cr and 8% Ni) were evaluated to be −447 mV and 0.08 µA/cm^2^, respectively, in an artificial saliva environment [40]. The potential and current density of AISI 316L stainless steel (18% Cr and 12% Ni) were measured to be −483 mV and 0.02 µA/cm^2^, respectively, in an artificial saliva environment [41]. Therefore, a relative increase in the current density and corrosion rate in a fluoride-containing environment is reasonable in the present work. Hence, it was confirmed that the carbon film deposited for 80 min at a substrate bias voltage of +50 V exhibited the best corrosion-resistant performance.

The experimental results revealed that when the substrate bias voltage was set at +5 V, the carbon films detached after soaking in the fluorine-containing environment for 16 h, regardless of the deposition time. Raman spectroscopy and TEM characterizations confirmed that the carbon films deposited under +5 V exhibited an amorphous carbon structure, which demonstrated poor stability in the fluorine-containing environment and failed to provide effective protection for the underlying archwire substrate. In contrast, when the deposition time was fixed at 80 min, the degree of film detachment decreased progressively with increasing substrate bias voltage, and no detachment was observed at +50 V. Furthermore, Raman and TEM analyses indicated that the carbon films deposited at +50 V exhibited a nanocrystalline graphene structure, suggesting enhanced corrosion resistance and the ability to effectively resist the aggressive effects of active fluorine species.

**Figure 8:**
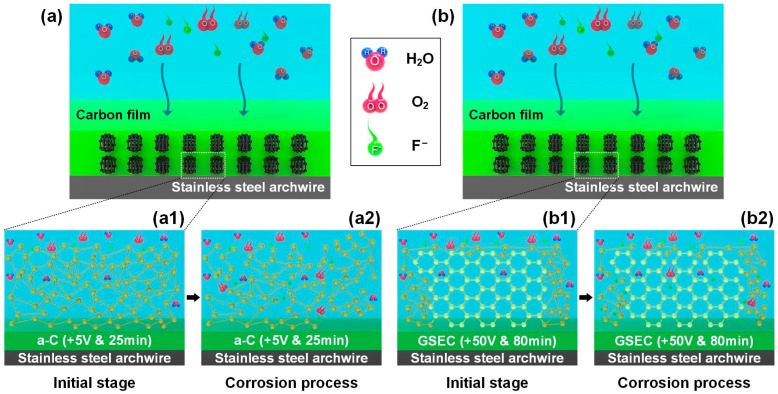
Schematic illustrations of the corrosion mechanism of the carbon-film-coated stainless steel archwire in a fluoride-containing environment. (**a**) Amorphous carbon film (+5 V & 25 min)-coated stainless steel archwire. (**a1**) Initial stage and (**a2**) corrosion process for amorphous carbon film. (**b**) Graphene-sheet-embedded-carbon film (+50 V & 80 min)-coated stainless steel archwire. (**b1**) Initial stage and (**b2**) corrosion process for GSEC film.

A hypothesis for the corrosion mechanisms of amorphous carbon-film-coated and GSEC-film-coated stainless steel archwires in fluoride-containing solutions is proposed, as schematically shown in Figure 8a,b, respectively. Specifically, Figure 8(a1,a2) illustrates that when soaking in a fluoride-containing environment, the amorphous carbon film (e.g., +5 V & 25 min) undergoes partial delamination due to the aggressive attack of active fluoride ions. This delamination re-exposed the underlying stainless steel archwire to the corrosive medium. Surface composition analysis revealed enrichment of F and O elements in the exposed regions, whereas their concentrations remained relatively low in the regions still covered by the intact carbon film. It was clarified that the amorphous carbon film provided a certain degree of protection as long as it remained intact. However, once delamination occurred, the protective capability was markedly reduced, allowing fluoride ions to further corrode surface of the stainless steel archwire. It was suggested that amorphous carbon film was inherently unstable in the fluoride-containing environment and was unable to provide sustained protection. On the contrary, Figure 8(b1,b2) demonstrates that the GSEC film exhibits a vertically aligned and densely packed microstructure on the stainless steel archwire surface, indicating superior structural integrity. Surface composition characterization confirmed that the levels of F and O elements on the GSEC film were minimal, suggesting strong adhesion even under fluoride exposure. While active fluoride ions may slightly affect the amorphous carbon regions at the edges, the nanocrystalline structure itself remained largely unaffected. It was revealed that the graphene nanocrystalline carbon film effectively acted as a barrier against fluoride ion penetration, therefore preventing severe corrosion of the stainless steel archwire in a fluoride-containing environment.

### 4.2. Wear Rate of the Carbon-Film-Coated Archwires

**Figure 9:**
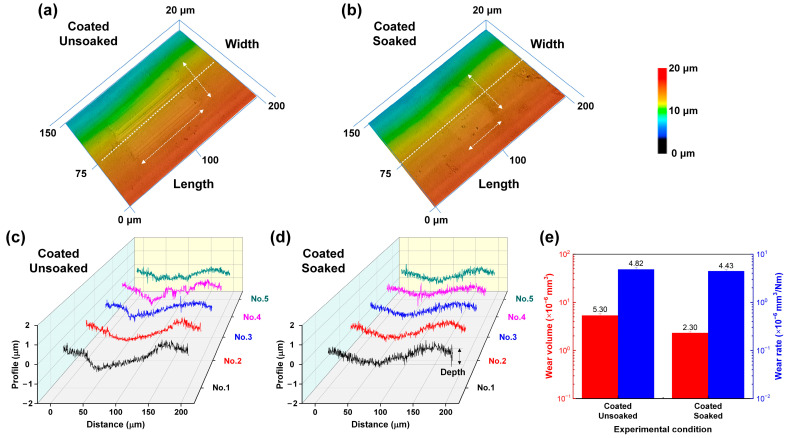
Calculation of the wear volume and specific wear rate of the unsoaked and soaked carbon-film-coated (i.e., +50 V & 80 min) stainless steel archwires after sliding against stainless steel brackets in an artificial saliva environment. (**a**) Three-dimensional optical image and corresponding (**b**) two-dimensional cross-sectional profile of the wear scar on the unsoaked/coated stainless steel archwire. (**c**) Three-dimensional optical image and corresponding (**d**) two-dimensional cross-sectional profile of the wear scar on the soaked/coated stainless steel archwire. (**e**) Calculated wear volume and specific wear rate.

To further investigate the wear resistance of carbon-film-coated archwires, 3D laser confocal microscopy was employed to systematically characterize the surface morphology of the worn surfaces on the stainless steel archwires. Figure 9a–d illustrates the three-dimensional images and corresponding two-dimensional cross-sectional profiles at five distinct points across the wear scar, measured along the white dashed lines. The wear depth and width of the carbon-film-coated archwires remained relatively consistent at approximately 0.65 μm (less than the thickness of the film, as shown in Figure S3) and 70 μm, respectively. However, a notable difference was observed in the wear scar length, where the length of the unsoaked coated archwire (100 mm) was approximately twice that of the soaked one (50 mm). As shown in Figure 9e, the wear volume of the unsoaked/coated archwire was 5.30 × 10^−6^ mm^3^, whereas that of the soaked/coated archwire was 2.30 × 10^−6^ mm^3^, with the former being around twice that of the latter. In terms of wear rate, the values were 4.82 × 10^−6^ mm^3^/Nm and 4.43 × 10^−6^ mm^3^/Nm, respectively, indicating a relatively small difference between the two experimental conditions. It was confirmed that soaking in a fluoride toothpaste mixed solution for 16 h did not significantly degrade the performance of the GSEC-film-coated stainless steel archwire, which retained its excellent wear resistance.

### 4.3. Low Friction Mechanism of the Carbon-Film-Coated Archwires

Carbon films with varying deposition times and substrate bias voltages were deposited on the surface of orthodontic archwires, followed by the immersion test in a fluorine-containing environment. The results revealed that the carbon film prepared under a deposition time of 80 min and a substrate bias voltage of +50 V exhibited superior corrosion resistance. Following 16 h of soaking, a friction and wear test was performed between the archwire and bracket. The experimental data indicated no significant change in the friction coefficient or wear rate before and after soaking. Specifically, the friction coefficient was measured at 0.06 prior to soaking and decreased to 0.04 after soaking, while the corresponding wear rates were 4.82 × 10^−6^ mm^3^/Nm and 4.43 × 10^−6^ mm^3^/Nm, respectively. Similarly, no statistically significant difference in static friction force was observed among the artificial saliva corrosion, mouthwash corrosion, and non-corrosion groups upon introduction of the DLC-coated metal bracket [42]. It was demonstrated that the graphene nanocrystalline carbon film retained excellent tribological performance even after exposure to a fluorine-containing environment, highlighting its dual advantages of corrosion resistance as well as anti-friction and anti-wear properties. Notably, it could effectively reduce the archwire–bracket friction coefficient from 0.17 to 0.06. Moreover, surface composition analysis of the carbon film-coated archwires (80-min deposition time and +50 V substrate bias) before and after immersion revealed no appreciable alteration in elemental composition, further confirming the structural stability and chemical inertness of the carbon film during the friction and wear process.

**Figure 10:**
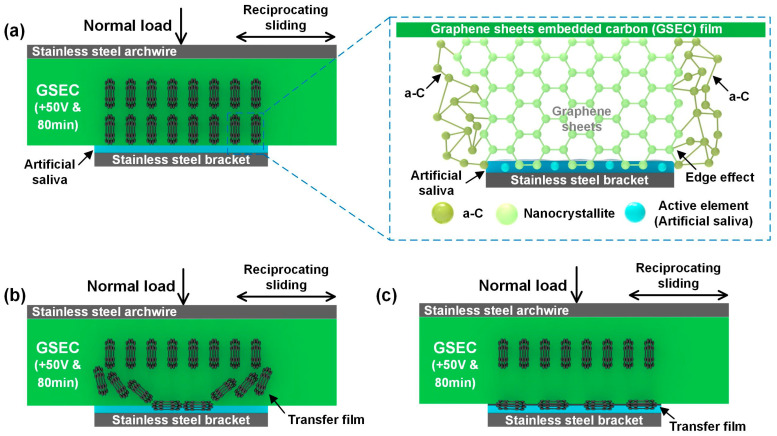
Schematic diagram of the low friction mechanism of the GSEC film (i.e., +50 V & 80 min) coated stainless steel archwire sliding against stainless steel bracket in artificial saliva environment. (**a**) Initial stage of the sliding contact combination of the stainless steel archwire–bracket. Inset illustration is the detailed atomic structure of the GSEC film. (**b**) Formation of the transfer film at the running-in stage. (**c**) Steady-state low friction coefficient of the GSEC-film-coated archwire with the covering of the transfer film rich in graphene sheets and adsorbed salivary layer.

A hypothesis for the low friction mechanism involving GSEC-film-coated stainless steel archwire and a stainless steel bracket is proposed, as schematically shown in Figure 10. The presence and/or formation of graphene nanocrystallites at the contact interfaces of amorphous carbon films, GSEC films, and graphene nanocrystalline carbon nitride (GNCN) films has been demonstrated to contribute significantly to achieving friction coefficients below 0.10 [32,33,35]. TEM analysis revealed that the graphene nanocrystalline carbon film exhibited a vertically oriented growth morphology on the surface of stainless steel archwire. Its densely packed microstructure contributed to excellent adhesion stability, enabling the film to maintain superior tribological performance even after soaking in a fluoride-containing environment. Figure 10a illustrates the contact interface between the archwire and bracket, where artificial saliva is continuously supplied and distributed across the contact zone. Under applied loading, intimate contact was established between the two components, with the archwire surface protected by a vertically aligned graphene nanocrystalline carbon film.

Figure 10b presents a schematic representation of the carbon film’s microstructure, highlighting how the edge effects of the densely packed nanocrystals promote the formation of an irregular amorphous carbon layer. During the reciprocating sliding between the archwire and bracket, a transfer film rapidly formed on the contact interface, leading to a significant reduction in the friction coefficient. Concurrently, the continuous supply of artificial saliva above the contact region facilitated the formation of an adsorbed lubricating film on the bracket surface [43,44], further enhancing the lubrication conditions, as illustrated in Figure 10c,d. The synergetic lubricating effects of the transfer film and the saliva-derived lubricating film resulted in a rapid decrease in friction coefficient, as shown in Figure 5b,c, which was subsequently maintained at a consistently low level throughout the entire sliding process. These findings not only elucidated the influence of substrate bias voltage on the stability of the carbon film but also highlighted its potential as a protective coating for metallic materials in corrosion-prone environments.

In this study, the GSEC film prepared under a substrate bias voltage of +50 V and a deposition duration of 80 min was found to exhibit excellent friction, wear, and corrosion resistance properties in a customized laboratory artificial saliva environment. These outstanding performances are favorable for the potential design of low-friction, light-force orthodontic appliances aimed at enhancing treatment efficiency and reducing clinical risks [6]. However, it is crucial to emphasize that the experimental results discussed above were obtained under controlled laboratory conditions (i.e., in vitro), which still differ significantly from the complex intraoral environment (i.e., in vivo). Furthermore, in practical clinical applications of carbon-based films, critical factors such as long-term stability, durability, and biocompatibility must be rigorously evaluated. These aspects require systematic investigation in future research.

## 5. Conclusions

In this study, carbon films were successfully fabricated on the working surfaces (width of 0.017 inch) of the stainless steel archwires under various deposition times (10 min to 80 min) as well as substrate bias voltages (+5 V to +50 V) using a self-designed MCECR plasma sputtering system with low energy electron irradiation. Structural analysis revealed that the carbon films deposited at the substrate bias voltages of +5 V and +10 V were amorphous carbon, whereas those fabricated at the substrate bias voltages of +20 V and +50 V were identified as graphene nanocrystalline carbon (i.e., GSEC) films. It was found that the area of carbon film detachment from the stainless steel archwire gradually decreased with increasing substrate bias voltage after soaking in a mixed solution of fluoridated toothpaste for 16 h. Notably, the GSEC film prepared at the substrate bias voltage of +50 V for 80 min exhibited no detachment at all, indicating excellent corrosion resistance. Electrochemical experiments further confirmed that the corrosion resistance of the fabricated carbon films improved significantly with higher substrate bias voltage. Moreover, the friction and wear performances of the GSEC films remained stable even after soaking in a fluorine-containing environment for 16 h. The friction coefficients before and after soaking were measured as 0.06 and 0.04, respectively, while the corresponding wear rates were 4.82 × 10^−6^ mm^3^/Nm and 4.43 × 10^−6^ mm^3^/Nm. Based on the characterization results, the corrosion resistance and friction mechanisms of the outstanding GSEC films were investigated. The nanocrystalline carbon film grew vertically on the archwire surface, and its dense microstructure contributed to its stability and resistance to attack by the active fluoride ions. Under the combined effects of applied load and shear stress, a transfer film formed rapidly on the sliding contact interface. The continuous supply of artificial saliva enhanced the lubrication properties of the adsorbed saliva film on the bracket. These two mechanisms synergistically led to a rapid reduction in the friction coefficient and its subsequent maintenance at a stable low level. Therefore, this work provides new insights into the application of GSEC films in friction and wear reduction as well as corrosion resistance, promoting the practical clinical application for utilizing the coated orthodontic stainless steel archwires.

## Figures and Tables

**Table 1:** Electrochemical corrosion parameters of uncoated and carbon-film-coated stainless steel archwires in a fluoride toothpaste mixed solution.

Substrate Bias Voltage (V)	Potential (mV/SCE)	Current Density (μA/cm^2^)	Corrosion Rate (mpy)
None	−303.1	0.336	62.81 × 10^−3^
+5	−226.3	0.229	34.40 × 10^−3^
+10	−200.8	0.253	37.97 × 10^−3^
+20	−159.5	0.084	12.63 × 10^−3^
+50	−135.1	0.072	10.74 × 10^−3^

## Data Availability

The original contributions presented in this study are included in the article/Supplementary Material. Further inquiries can be directed to the corresponding author.

## Supplementary Materials

The following supporting information can be downloaded at https://www.mdpi.com/article/10.3390/nano15211615/s1: Figure S1: Photograph of the customized electron cyclotron resonance plasma sputtering apparatus. Specifically, 1 is left coil, 2 is middle coil, 3 is right coil, 4 is main vacuum chamber, 5 is pre-vacuum chamber, 6 is program control panel, 7 is substrate holder, 8 is voltage and current control panel, and 9 is the microwave generator; Table S1: Experimental conditions of the carbon films deposition; Figure S2: Optical images of the wear tracks on the stainless steel brackets after sliding against different archwires in an artificial saliva environment. (a) Uncoated/unsoaked archwire. (b) Coated/unsoaked archwire. (c) Coated/soaked archwire. The wear tracks, which are primarily located at the edges of the slot surfaces, are indicated by red dotted rectangles; Figure S3: Cross-sectional profile of the GSEC film fabricated under a substrate bias voltage of +50 V and a deposition time of 80 min. The calculated film thickness was determined to be 0.66 mm.
