# Supplementary material for: Low-Friction and Corrosion-Resistant Orthodontic Stainless Steel Archwires with Functional Carbon Films
Source: Nanomaterials (Basel). 2025 Oct 23;15(21):1615. doi: 10.3390/nano15211615 (PMC12609674; doi:10.3390/nano15211615)
Supplement: Supplementary file 1 [file nanomaterials-15-01615-s001.zip › nanomaterials-3925450-supplementary.pdf]

Supplementary Material

Low-Friction and Corrosion-Resistant Orthodontic Stainless Steel Archwires with Functional Carbon Films

Pengfei Wang *, Minghui Hao and Shiqi Cheng

Institute of Nanosurface Science and Engineering (INSE), State Key Laboratory of Radio Frequency Heterogeneous Integration, College of Mechatronics and Control Engineering, Shenzhen University, Shenzhen 518060, China

*Corresponding author. E-mail address: wangpf@szu.edu.cn (Wang, P.F.)

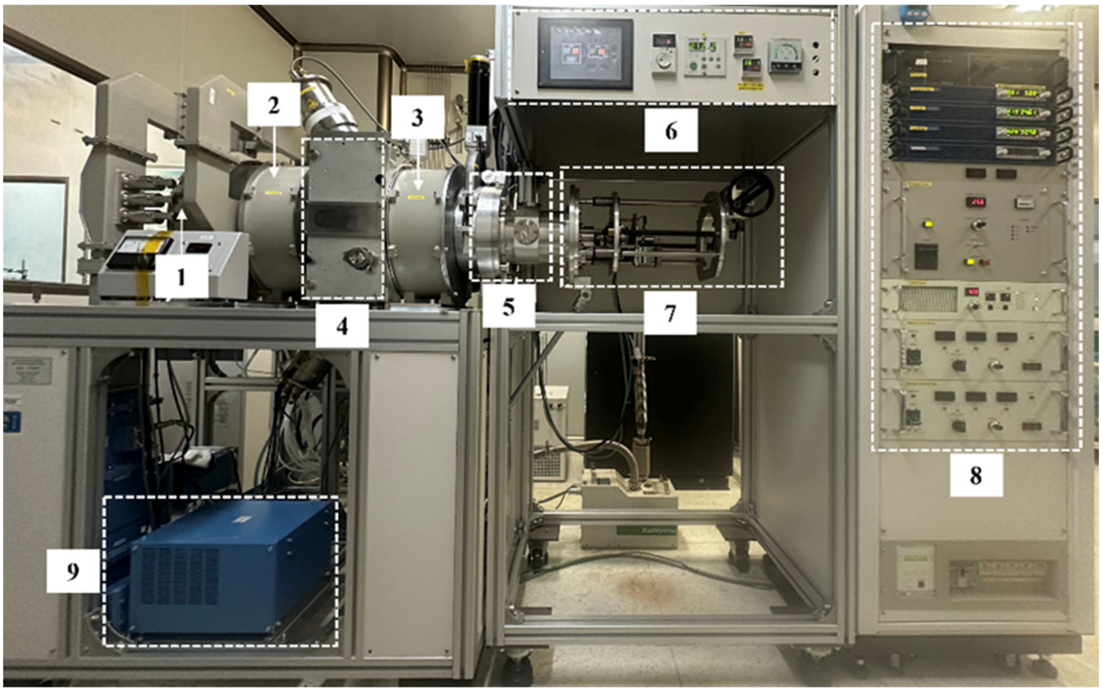

Figure S1. Photograph of the customized electron cyclotron resonance plasma sputtering apparatus. Specifically, 1 is left coil, 2 is middle coil, 3 is right coil, 4 is main vacuum chamber, 5 is pre-vacuum chamber, 6 is program control panel, 7 is substrate holder, 8 is voltage and current control panel, and 9 is the microwave generator.

Table S1. Experimental conditions of the carbon films deposition.

Deposition parameters	Values
Gas pressure (Pa)	0.1
Microwave power (W)	256
Target bias voltage (V)	−500
Coil current (A)	Left: 40 / Middle: 40 / Right: 48
Substrate bias voltage (V)	+5 / +10 / +20 / +50
Deposition time (min)	25 / 35 / 50 / 80

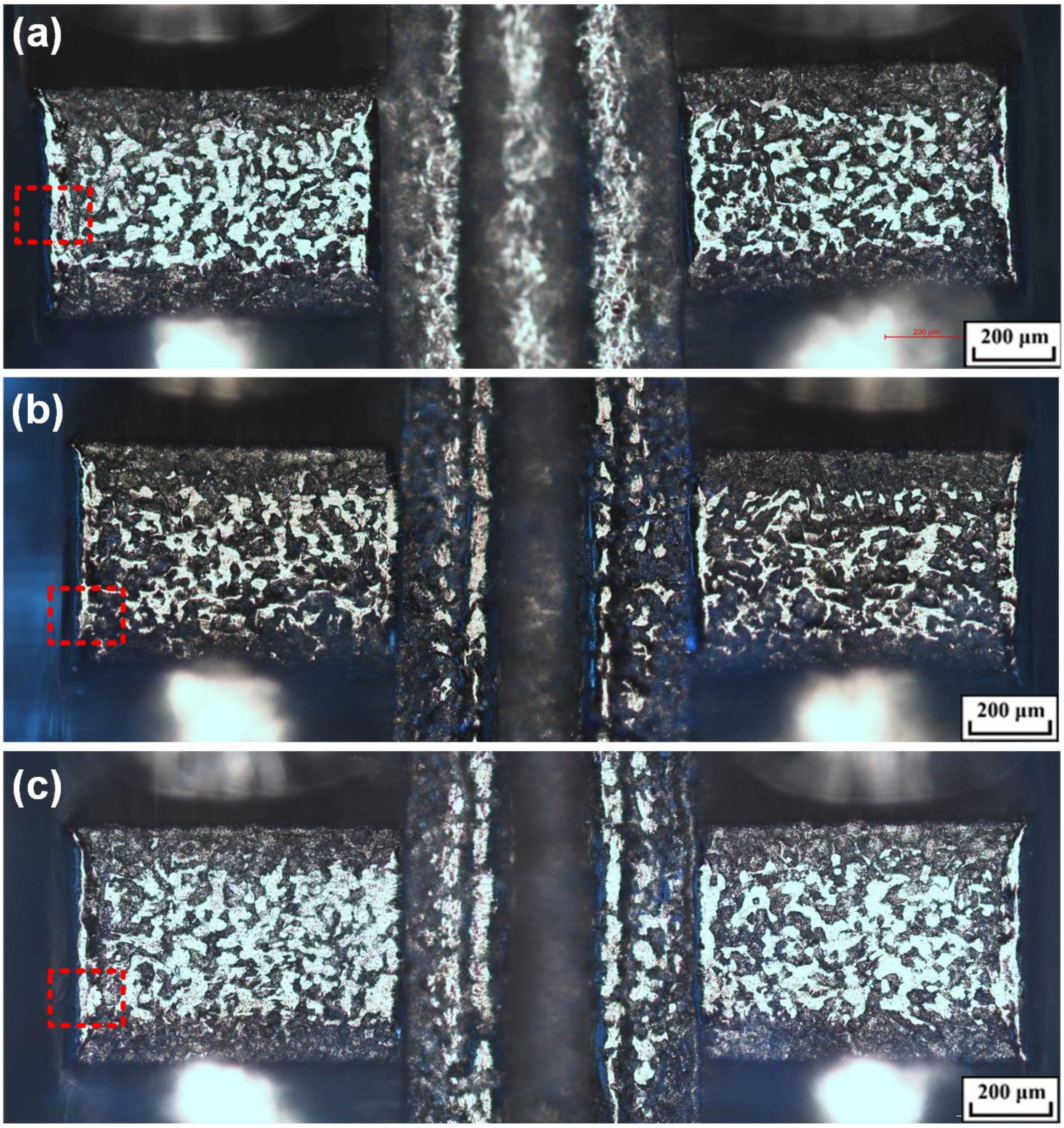

Figure S2. Optical images of the wear tracks on the stainless steel brackets after sliding against different archwires in an artificial saliva environment. (a) Uncoated/unsoaked archwire. (b) Coated/unsoaked archwire. (c) Coated/soaked archwire. The wear tracks, which are primarily located at the edges of the slot surfaces, are indicated by red dotted rectangles.

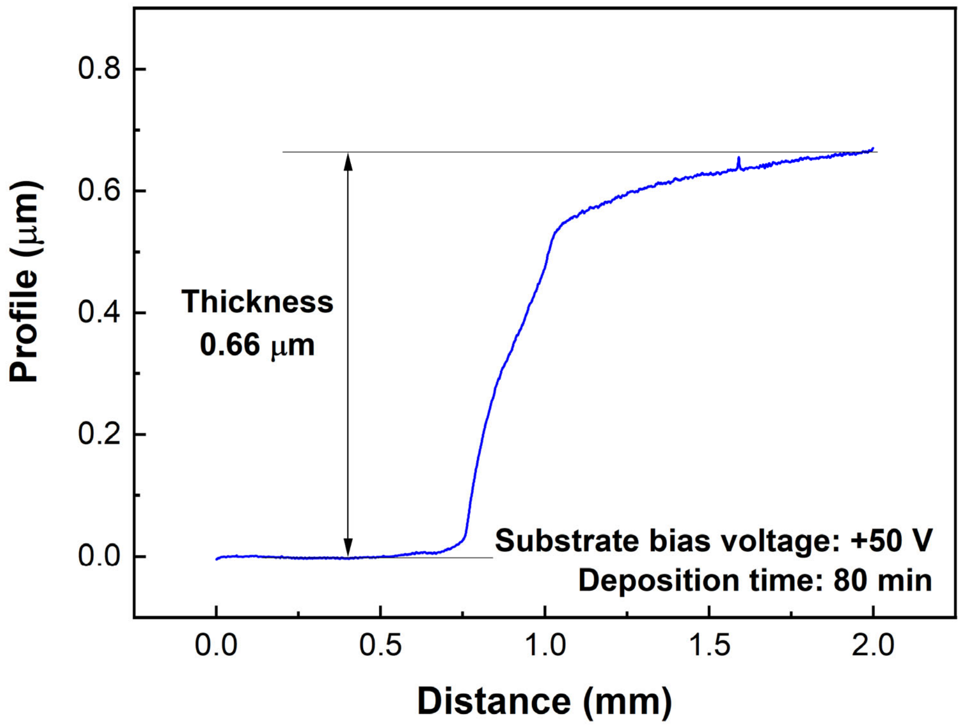

Figure S3. Cross-sectional profile of the GSEC film fabricated under a substrate bias voltage of +50 V and a deposition time of 80 min. The calculated film thickness was determined to be 0.66 μm.
